# Precision Position Control of a Voice Coil Motor Using Self-Tuning Fractional Order Proportional-Integral-Derivative Control

**DOI:** 10.3390/mi7110207

**Published:** 2016-11-14

**Authors:** Syuan-Yi Chen, Chen-Shuo Chia

**Affiliations:** Department of Electrical Engineering, National Taiwan Normal University, Taipei 106, Taiwan; 40275022h@ntnu.edu.tw

**Keywords:** proportional-integral-derivative control, voice coil motor, differential evolution, fractional order

## Abstract

The object of this study is to develop a self-tuning fractional order proportional-integral-derivative (SFOPID) controller for controlling the mover position of a direct drive linear voice coil motor (VCM) accurately under different operational conditions. The fractional order proportional-integral-derivative (FOPID) controller can improve the control performances of the conventional integer order PID controller with respect to the additional fractional differential and integral orders; however, choosing five interdependent control parameters including proportional, integral, and derivative gains, as well as fractional differential and integral orders appropriately is arduous in practical applications. In this regard, the SFOPID controller is proposed in which the five control parameters are optimized dynamically and concurrently according to an adaptive differential evolution algorithm with a high efficiency adaptive selection mechanism. Experimental results reveal that the SFOPID controller outperforms PID and FOPID controllers with regard to the nonlinear VCM control system under both nominal and payload conditions.

## 1. Introduction

The linear voice coil motor (VCM) is a direct drive and hysteresis-free device used for providing highly accurate linear motion. The linear VCMs utilize a magnet field generated by permanent magnets in connection with a coil of wires to produce an electric driving force for high precision position control [1,2,3]. Since the mass of the moving coil is typically low, the speed and acceleration of the VCM are very high and the settling time is very short. Moreover, because the inductance of a VCM is typically low, the resulting low electrical time constant enables the VCM to have a very fast response and bandwidth. Besides, the direct drive features benefits for the VCM such as zero cogging, zero backlash, smooth motion at low speeds, and limitless resolution [1,2,3,4,5]. Therefore, VCMs have been widely applied to various small range positioning applications [6,7,8,9]. However, because the dynamic of the VCM is nonlinear and time-varying due to the variations of motor parameters and mechanical friction force, many advanced control strategies have been developed to control the VCM with high control performances such as optimal control [4], sliding-mode control [5,10], proportional-integral-derivative (PID) control [8], neural network control [11], and robust control [12].

The PID control scheme is generally preferred in many industrial and academic applications [13,14]. However, it is difficult to obtain satisfactory control performances in highly nonlinear and time-varying systems for the conventional PID controller due to its linear structure, constant control gains and limited degrees of freedom. To address this drawback, the integer order PID controller has been generalized to a fractional order PID (FOPID) controller by the addition of fractional integral and derivative orders [15,16,17,18,19]. Compared with the conventional PID control, the FOPID control can achieve better robustness and control performance levels with respect to the two well-defined fractional orders [17,18]. However, an optimal set for the control parameters cannot be obtained easily, which includes proportional (P) gain (*k_p_*), integral (I) gain (*k_i_*), derivative (D) gain (*k_d_*), the fractional order of the integral (α), and the fractional order of the derivative (β). Therefore, many strategies have been proposed to tune the control parameters of the PID and FOPID controllers automatically, such as the flat phase method [15], the frequency domain-based design [16], optimal tuning methods [17,18], and evolutionary algorithm (EA) [19,20]. 

EAs have been widely applied to solve real-world problems, such as manufacturing [21,22], and structure design [23] because they can generate high-quality solutions to multi-dimensional optimization and search problems by relying on bio-inspired operations. Among various EAs, the differential evolution (DE) algorithm has been used in a wide range of application fields [24,25,26,27,28,29,30]. In DE, three crucial control parameters including population size (*NP*), mutation factor (*F*), and crossover rate (*CR*), significantly affect the optimization performance [31,32]. Therefore, to effectively solve a specific optimization problem, a time-consuming trial-and-error search procedure for choosing the best parameter values is always required. To address this problem, many variants of DE have been developed to improve the adaptiveness, efficiency, and performance of the conventional DE [30,31,32,33].

The object of this study is to develop a new self-tuning FOPID (SFOPID) control strategy for controlling the mover position of a direct drive VCM precisely. To derive the control parameters of the SFOPID controller optimally, an adaptive DE (ADE) algorithm is adopted to tune the control parameters online. Therefore, not only tracking performances can be improved but also trivial trials for the control parameters can be discarded. Furthermore, stable control performances during the control process can be ensured. Experimental results with performance measures are given to verify the effectiveness and high-precision position control performance levels of the proposed SFOPID controller. The rest of this study is organized as follows: the operational principle and dynamics of the linear VCM are described in Section 2; the ADE algorithm used for optimizing the control parameters is presented in Section 3; the developed FOPID control and SFOPID control on the basis of fractional calculus are given in Section 4, and the experimental results and conclusion are given in Section 5 and Section 6, respectively.

## 2. Linear Voice Coil Motor

**Figure 1:**
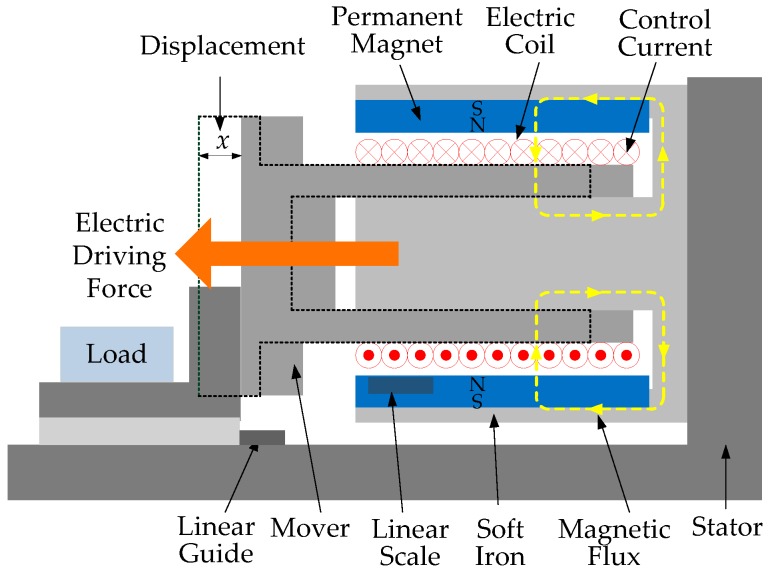
Structure of a voice coil motor (VCM).

The structure of a linear circular moving coil-type VCM, which consists of a moving electric coil winding assembly and a stationary permanent magnet field assembly within a soft iron shell, is illustrated in Figure 1. As can be seen in Figure 1, the current flowing through the coil interacts with the permanent magnetic field and then generates a force perpendicular to the directions of the magnetic field and the current based on the Lorentz electric force principle. The generated electric driving force is proportional to the product of the magnetic field and the drive current. Thus, a positive applied voltage causes the voice coil to move in one direction on a linear guide; while a reversed voltage causes it to move in the opposite direction. Therefore, VCM are bidirectional actuators with similar behavior in both directions. Furthermore, it is noted that the direct coupling of the motor to the load results in high accuracy operational features. In addition, a high resolution linear scale provides high precision mover position information for closed-loop control. In this way, the acceleration, velocity and position of the mover can be controlled effectively.

To describe the dynamic of the VCM, the state-space model is given as follows [3]:

(1) $$\dot{x}=Ax(t)+Bu(t)+MF(t),\;y(t)=x_{1}(t)\;x=\left[\begin{array}{l} x_{1}\\ x_{2} \end{array}\right]=\left[\begin{array}{l} x\\ \dot{x} \end{array}\right]\;A=\left[\begin{array}{cc} 0 & 1\\ -\frac{K_{k}}{m+M} & -\frac{K_{b}}{m+M} \end{array}\right],B=\left[\begin{array}{c} 0\\ \frac{CK_{i}}{m+M} \end{array}\right],M=\left[\begin{array}{c} 0\\ \frac{-1}{m+M} \end{array}\right]$$

where *u*(*t*) is the control voltage, *C* is the linear gain of the current amplifier, i.e., *i*(*t*) = *Cu*(*t*), *x* is the mover displacement, *M* and *m* are the masses of the mover and payload, respectively, *K_i_*, *K_k_*, and *K_b_* are the force-current coefficient, equivalent elastic load coefficient, and equivalent damping coefficient of VCM, respectively, and *F*(*t*) is the total unmodeled load force. As seen in Equation (1), though the VCM can be expressed by a state-space model, the practical control characteristics of the VCM are nonlinear because the coefficients *K_i_*, *K_k_*, and *K_b_* may vary according to the changes in operating temperature and period. Moreover, the time-varying unmodeled load force *F*(*t*) is not easy to measure exactly. In this regard, designing a model-free control strategy for the VCM control system is essential in practical applications.

## 3. Adaptive Differential Evolution Algorithm

**Figure 2:**
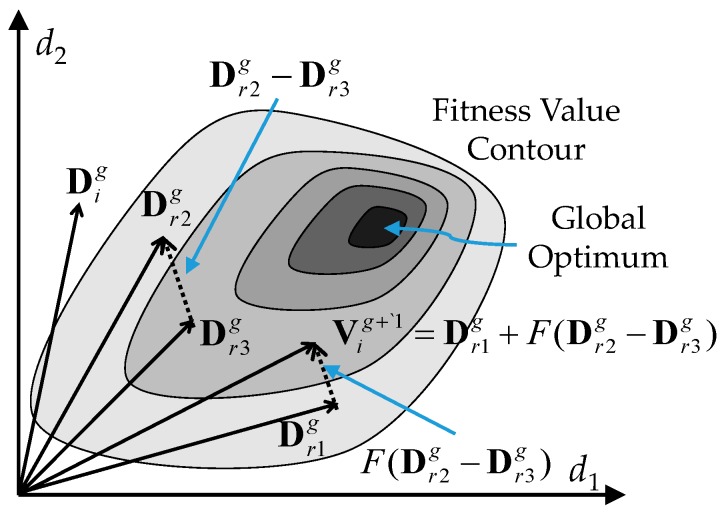
Example of a two-dimensional fitness contour showing the process for generating a mutant vector $V_{i}^{g+1}$.

DE is a vector population based stochastic optimization method that is able to optimize an objective function effectively. An unconstrained optimization problem can be stated as follows:

(2) $$\mathrm{Find}\;D=[d_{1},\;d_{2},...\;d_{j},...\;,d_{\Phi }]\text{ which maximizes }f(D)$$

where **D** is an Φ-dimensional vector in the search space, *j* = 1, 2, …, Φ, and *f*(**D**) is an objective function. Regarding the conventional DE algorithm, four steps including population, mutation, crossover and selection, are explained in the following [24,25,26,27,28,29]:

1. Population: In the initial population step, the DE algorithm generates the initial individual target vector $D_{i}^{g}$ randomly as follows:
  (3) $$D_{i}^{g}=[d_{i,1}^{g},\;d_{i,2}^{g},...\;d_{i,j}^{g},...\;,d_{i,\Phi }^{g}]$$
  where *i* = 1, 2,…, *NP*, in which *NP* is the population size; and *g* represents the *g-*th generation of the population.
2. Mutation: There are several techniques for the mutation of target vector $D_{i}^{g}$. Commonly, three individual target vectors, $D_{r1}^{g}$, $D_{r2}^{g}$, and $D_{r3}^{g}$ among the population are randomly selected to generate the mutant vector $V_{i}^{g+1}$ according to the following mutation mechanism:
  (4) $$V_{i}^{g+1}=D_{r1}^{g}+F(D_{r2}^{g}-D_{r3}^{g})$$
  where *r*1 ≠ *r*2 ≠ *r*3 ≠ *i*, and *F* is a constant mutation factor. In general, the small constant mutation factor *F* may lead to premature convergence and even convergence at a local optimal solution, whereas a large one may result in poor efficiency with long convergence time. Therefore, an adaptive selection mechanism for the dynamic mutation factor *F* is adopted to address the mentioned problems as follows [31]:
  (5) F=φ×ζ×λ
  where φ is a uniformly distributed random variable between (0, 1), ζ is an momentum weight, and λ is an adaptive factor defined as
  (6) $$\zeta =\sqrt{\frac{g_{\max}-g_{\mathrm{now}}}{g_{\max}}}$$
  where *g*_max_ is the maximum generation number, and *g*_now_ is the current generation number. Besides, the adaptive factor λ is designed to balance the global exploration and local search abilities as follows:
  (7) $$\lambda =\left\{ \begin{array}{l} \lambda_{b}\geq 1,\;\mathrm{if}\;q\leq q_{d}\\ \lambda_{s}<1,\;\mathrm{if}\;q>q_{d} \end{array}\right.$$
  where *q_d_* is the threshold of the acceptable improvement rate, λ*_b_* and λ*_s_* are the big and small step sizes, respectively, and *q* is the improvement rate of the fitness value in the specific generation. For the target vector $D_{i}^{g}$, the improvement rate *q* can be obtained as follows:
  (8) $$q_{i}^{g}=\frac{f(D_{i}^{g})-f(D_{i}^{g-N_{q}})}{f(D_{i}^{g-N_{q}})}$$
  where *N_q_* < *g* is a generation number used for evaluating the improvement rate of the fitness value*.* According to Equations (7) and (8), the adaptive factor λ is reduced to a small step size λ*_s_* to strengthen the local search ability when the present fitness value has a favorable improvement. Conversely, the adaptive factor λ is enlarged to a big step size λ*_b_* to strengthen the global exploration ability when the present fitness value has an unacceptable improvement. Figure 2 shows the mutation operation via a two-dimensional example.
3. Crossover: The most common crossover strategy is uniform crossover in which the individual target vector $D_{i}^{g}$ is crossed over with its mutant vector $V_{i}^{g+1}$ for generating the new trial vector $U_{i}^{g+1}$ as follows:
  (9) $$u_{i,j}^{g+1}=\{\begin{array}{cc} \;v_{i,j}^{g+1}, & \mathrm{if}\;rand_{j}\leq CR\\ d_{i,j}^{g}, & \mathrm{if}\;rand_{j}>CR \end{array}$$
  where $u_{i,j}^{g+1}$, $v_{i,j}^{g+1}$, and $d_{i,j}^{g}$ are the *j-*th elements of the vectors $U_{i}^{g+1}$, $V_{i}^{g+1}$, and $D_{g}^{i}$, respectively; *rand_j_* is a uniformly distributed random variable between (0,1); and *CR* is a predesigned constant crossover rate.
4. Selection: The final step in DE algorithm is the selection of the better individual for maximizing the objective function *f*(**D**), as shown in Equation (2). The selection process uses a simple replacement of the original target vector $D_{g}^{i}$ with the obtained new trial vector $U_{i}^{g+1}$if the latter has a better fitness value. The better individual vector is then selected as a new target vector $D_{i}^{g+1}$ for the next generation as follows:
  (10) $$D_{i}^{g+1}=\left\{ \begin{array}{l} U_{i}^{g+1},\;\mathrm{if}\;f(U_{i}^{g+1})\geq f(D_{i}^{g})\\ D_{i}^{g},\;\mathrm{otherwise} \end{array}\right.$$

Repeat Steps 1–4 until the best fitness value is achieved or a preset count of the generation number is reached.

## 4. Proposed Control Methods

### 4.1. Fractional Order Calculus

Fractional order calculus is a generalization of integer order integration and differentiation. Let symbol *_a_*$D_{t}^{\lambda }$ denotes the fundamental fractional order operator as follows [34,35]:

(11) Dλ≡Dtλa={dλdtλ,1,∫at(dτ)−λ,ℜ(λ)>0ℜ(λ)=0ℜ(λ)<0

where *a* and *t* are the limits of the operation; λ is the fractional order of the operator, which can be a real or complex number; and ℜ(λ) denotes the real part of λ. The commonly used definitions for the fractional operators are the Caputo, Grunwald-Letnikov (GL) and Riemann-Liouville (RL) definitions. First, the λ-th-order Caputo definition is given as follows [36]:

(12) Dtλaf(t)={1Γ(m−λ)∫at(t−τ)m−λ−1f(m)(τ)dτ, m−1<λ<mdmdtmf(t), λ=m

where *m* is the first integer and Γ(·) is a Gamma function. Moreover, the λ-th-order GL definition is given by [35] as follows:

(13) Dtλaf(t)=limh→01hλ∑j=0t−ah(−1)j(λj)f(t−jh)

where *h* is the step-size in computation, and

(14) $$\left(\begin{array}{l} \lambda \\ j \end{array}\right)=\frac{\Gamma (\lambda +1)}{\Gamma (j+1)\cdot \Gamma (\lambda -j+1)}$$

Furthermore, the λ*-*th-order RL definition is provided by [35] as follows:

(15) Dtλaf(t)=1Γ(r−λ)drdtr∫at(t−τ)r−λ−1f(τ)dτ,r−1<λ<r

where *r* is the first integer. For convenience, Laplace transformation is usually used to describe the fractional integral and derivative operations. The Laplace transforms for the fractional calculus shown in Equations (12)–(15) under zero initial conditions are given as follows [35]:

(16) L  {Dt±λ0f(t)}=∫0∞e−stDt±λ0f(t)=s±λF(s)

where *s* = *j*ω denotes the Laplace operator and *F*(*s*) is the Laplace transform of the function *f*(*t*) with condition *a* = 0. Obviously, the integral and derivative operations with fractional orders have more degrees of freedom than those with integer orders. 

### 4.2. Fractional Order Proportional-Integral-Derivative Control

**Figure 3:**
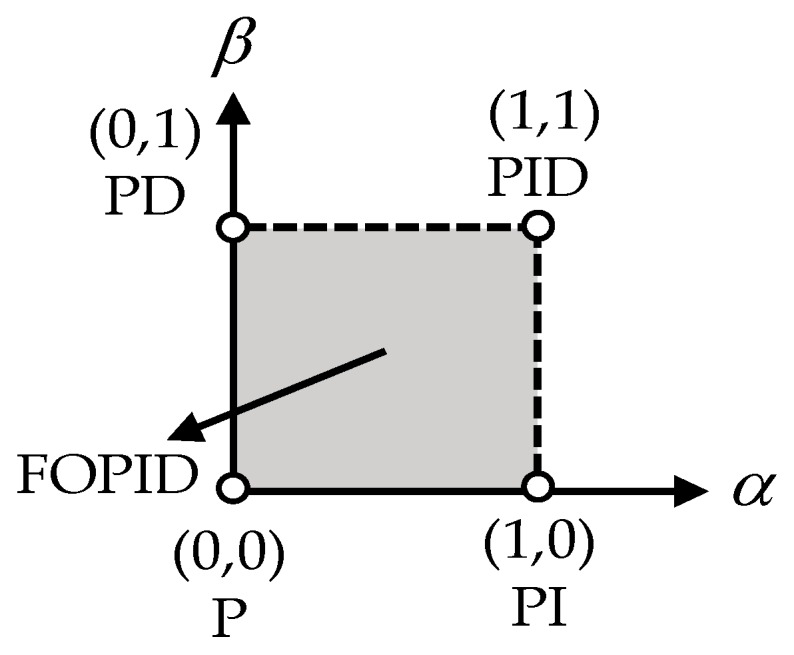
Comparison of the P, PI, PD, PID, and fractional order PID (FOPID) controllers.

To improve the control performance levels and robustness of the conventional integral order PID controller, the FOPID controller with fractional order integration and derivation parts is given as follows:

(17) u(t)=kpe(t)+kDt−αie(t)+kDtβde(t)

where *e*(*t*) is the tracking error between the reference trajectory *x_d_* and the practical mover position *x*; *u*(*t*) indicates the control voltage applied in Equation (1). Thus, different control systems can be obtained according to the proper designs of the parameters α and β. Specifically, the P controller, PI controller, PD controller, and PID controller can be engendered under the selections (0,0), (1,0), (0,1), and (1,1), as shown in Figure 3. They justify that all these typical controllers are the special cases of the FOPID controller [16]. In this regard, the FOPID controller expands from point representation to a plane to provide more freedoms of control parameters. The continuous transfer function of the FOPID controller as shown in (17) can be obtained according to the Laplace transformation as:

(18) $$G_{c}(s)=\frac{U(s)}{E(s)}=k_{p}+k_{i}s^{-\alpha }+k_{d}s^{\beta }$$

where *U*(*s*) and *E*(*s*) are the control voltage *u*(*t*) and tracking error *e*(*t*) in s-domain, respectively. As seen from (17) and (18), the integral operator $D_{t}^{-\alpha }e$ shown in (17) can be considered as a low-pass filter of variable *e*. When *α* is selected properly, the steady-state error can be reduced effectively. On the other hand, the differential operator $D_{t}^{\beta }e$ can be considered as a high-pass filter of variable *e*. The response speed can be increased if a suitable β is chosen. Therefore, compared with the conventional integer order PID controller, the FOPID controller can achieve better robustness and control performance levels with respect to the two well-defined fractional integral and differential orders. However, to achieve superior position control performances for the FOPID controller, three control gains {*k_p_*, *k_i_*, *k_d_*} and two fractional orders {α, β} should be optimally designed for a given system. 

### 4.3. Self-Tuning Fractional Order Proportional-Integral-Derivative Control

To achieve superior position control performances, three control gains {*k_p_*, *k_i_*, *k_d_*} and two fractional orders {α, β} are optimized in the proposed SFOPID controller by using the ADE algorithm. The most crucial step in applying the ADE algorithm is to choose the objective function for evaluating the fitness value of each target vector. In this study, an absolute tracking error is employed to design the objective function. Thus, the optimization problem arising in this study can be expressed by rewriting Equation (2) as follows:

(19) $$\mathrm{Find}\;D=[k_{p},\;k_{i},k_{d},\alpha ,\beta ]\text{ which maximizes }f(D)=\frac{1}{\varepsilon +\left|e\right|}$$

where the target vector **D** consists of five control parameters; ε is a small positive constant. According to the design of the object function shown in Equation (19), the optimal parameters *k_p_*, *k_i_*, *k_d_*, α, and β can be obtained dynamically to minimize the tracking error *e* via the ADE algorithm. 

**Figure 4:**
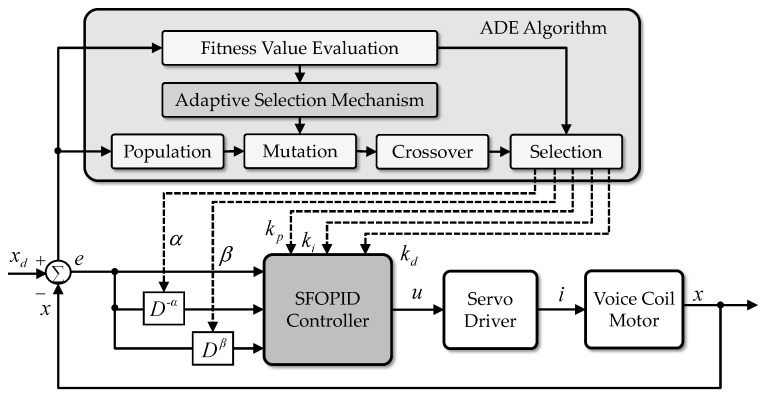
Control diagram of the VCM control system using the proposed self-tuning FOPID (SFOPID) controller with adaptive differential evolution (ADE) algorithm.

A block diagram of the VCM control system with the SFOPID controller is shown in Figure 4. In this control scheme, the SFOPID controller and ADE algorithm are utilized to determine the control voltage *u*(*t*) and optimize the control parameters *k_p_*, *k_i_*, *k_d_*, α and β, respectively. In the beginning, several target vectors are selected randomly within the specific searching ranges. Then, each vector **D** is applied to the SFOPID controller sequentially and the corresponding tracking performances are evaluated respectively. Finally, the vector with the highest fitness value is selected for the VCM control system.

### 4.4. Digital Implementation for Fractional Order Calculus

In this study, a trapezoidal (Tustin) discretization operator is applied for the approximation of fractional order calculus. First, a generating function is defined as follows [34,37]:

(20) $${\left(\omega (z^{-1})\right)}^{\lambda }={\left(\frac{2}{T}\right)}^{\lambda }{\left(\frac{1-z^{-1}}{1+z^{-1}}\right)}^{\lambda }={\left(\frac{2}{T}\right)}^{\lambda }\underset{n\to \infty }{\lim}\frac{A_{n}(z^{-1},\lambda )}{A_{n}(z^{-1},-\lambda )}$$

for obtaining the coefficients and the form of the approximation in which *T* is the sample period and *A_n_* can be derived recursively by [34,37]:

(21) $$A_{o}(z^{-1},\lambda )=1,A_{n}(z^{-1},\lambda )=A_{n-1}(z^{-1},\lambda )-c_{n}z^{n}A_{n-1}(z,\lambda )$$

and

(22) $$c_{n}=\left\{ \begin{array}{l} \lambda /n,\;n\;\mathrm{is}\;\mathrm{odd};\\ 0\;,\;n\;\mathrm{is}\;\mathrm{even}. \end{array}\right.$$

It is noted that only the recursive formula for positive λ is considered here to simplify the presentation. Now, the Laplace operator as shown in Equation (16) can be approximated by any given order *n* for digital implementation as

(23) $$S^{\lambda }\approx {\left(\frac{2}{T}\right)}^{\lambda }\frac{A_{n}(z^{-1},\lambda )}{A_{n}(z^{-1},-\lambda )}$$

## 5. Experimentation

### 5.1. Experimental Setup

**Figure 5:**
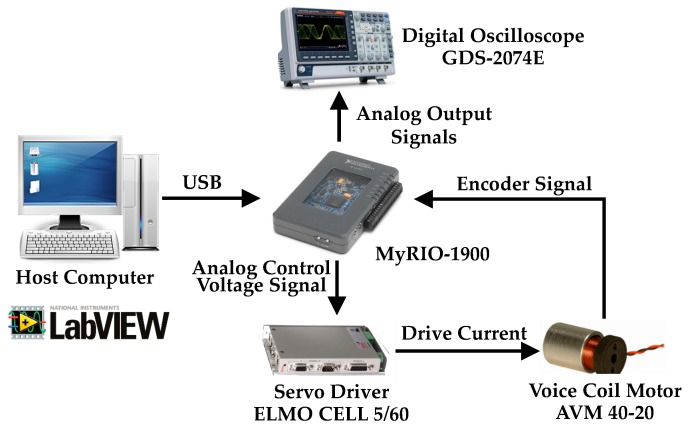
Experimental setup of the voice coil motor (VCM) control system.

The experimental setup of the VCM control system is composed of a VCM AVM 40-20 (Akribis Systems Pte. Ltd., Singapore), an Elmo Cello 5/60 servo driver (Elmo Motion Control Ltd., Petach-Tikva, Israel), a host computer with LabVIEW programming software, a National Instruments (NI) MyRIO-1900 (National Instruments, Austin, TX, USA) embedded control platform, and a digital oscilloscope GDS-2074E (Good Will Instrument Ltd., New Taipei City, Taiwan). The system is shown in Figure 5. The NI MyRIO-1900 providing analogy output and encoder interface in a compact device is the control core and connects to the host computer over USB port. In this study, the MyRIO-1900 first calculates the mover position from the encoder interface and then calculates the tracking error and its derivative. Subsequently, the control voltage *u* is determined with 1-kHz execution frequency by means of the PID, FOPID, and SFOPID controllers. Finally, the derived control signals are sent to the servo driver through a 12-bit resolution digital-to-analog conversion.

### 5.2. Performance Measures and Comparison

To compare the distinct control properties of the various controllers, three performance indexes including the maximum tracking error $P_{M}$, the average tracking error $P_{A}$, and the standard deviation of the tracking error $P_{S}$ are measured as follows:

(24) $$P_{M}=\underset{I}{\max}(\left|e(I)\right|)$$

(25) $$P_{A}=\frac{\sum_{I=1}^{R}\left(\left|e(I)\right|\right)}{R}$$

(26) $$P_{S}=\sqrt{\sum_{I=1}^{R}\frac{{\left(\left|e(I)\right|-P_{A}\right)}^{2}}{R}}$$

where *I* and *R* are the current and total iteration numbers during the control process. Moreover, two test conditions are provided in this study, which are the nominal case (Case 1) and the payload case (Case 2). In Case 2, one load with a 4.2-kg weight was added on the mover.

### 5.3. Experimental Results

In the experimentation, the constant parameters for the PID controller were chosen as *k_p_* = 16, *k_i_* = 30, and *k_d_* = 2. Besides, the constant parameters for the FOPID controller were chosen as *k_p_* = 10, *k_i_* = 25, *k_d_* = 1.5, α = 0.5, and β = 0.5. In this study, ninth-order approximation was implemented for fractional order calculus, i.e., *n* = 9. In this study, the control parameters *k_p_*, *k_i_*, *k_d_*, α and β were selected on the basis of several trials to achieve the favorable transient response considering the occurrence of uncertainties and the requirement of steady-state stability. However, it is difficult to choose all five control parameters simultaneously. In this study, small values were selected for the control parameters in the first instance. Afterward, they were further tuned by trial-and-error procedures to achieve the favorable transient control performance levels regarding the steady-state stability. However, it cannot be ensured that the PID and FOPID controllers can achieve the best control performances by adopting the manually designed control parameters. 

**Figure 6:**
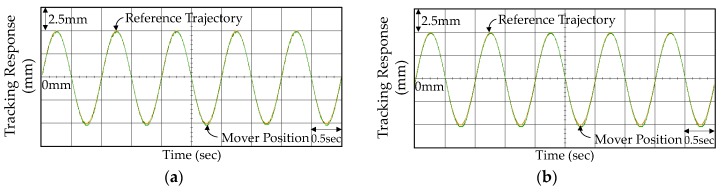

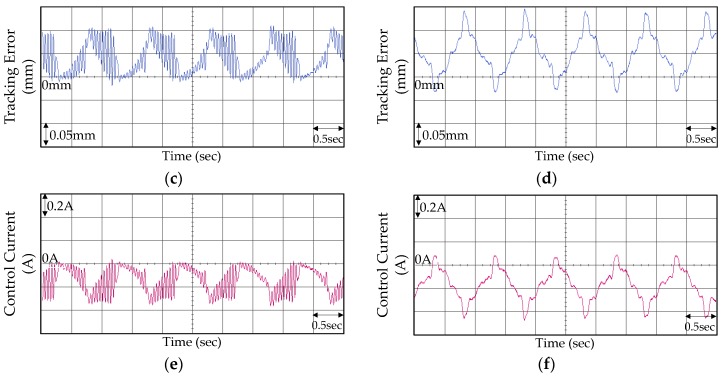
Experimental results of the VCM control system using the PID controller: (**a**) tracking response in Case 1; (**b**) tracking response in Case 2; (**c**) tracking error in Case 1; (**d**) tracking error in Case 2; (**e**) control current in Case 1; and (**f**) control current in Case 2.

**Figure 7:**
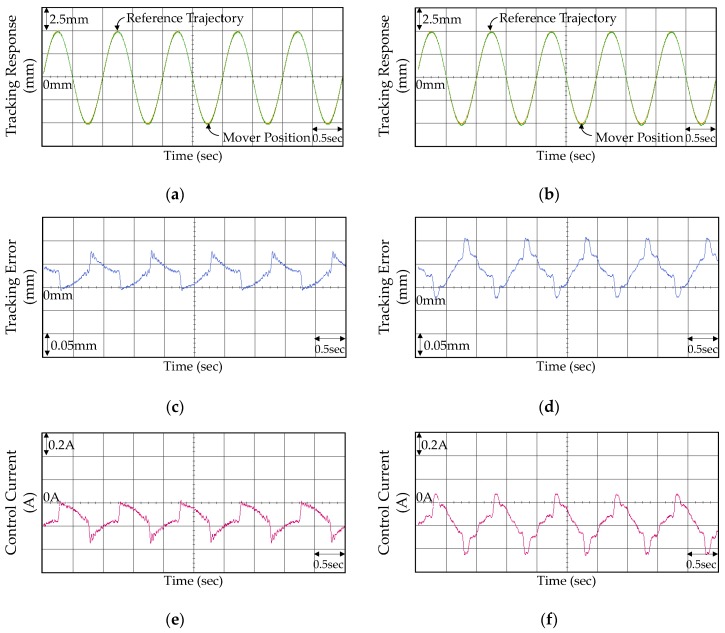
Experimental results of the VCM control system using the FOPID controller: (**a**) tracking response in Case 1; (**b**) tracking response in Case 2; (**c**) tracking error in Case 1; (**d**) tracking error in Case 2; (**e**) control current in Case 1; and (**f**) control current in Case 2.

The experimental results including tracking responses, tracking errors, and control currents of the VAM control system using the PID controller in Case 1 and Case 2 are shown in Figure 6. From the experimental results shown in Figure 6a,b, the mover of the VAM was controlled by the PID controller to track the reference trajectory certainly. However, the tracking responses were not favorable due to the vibrant tracking errors for Case 1 and large steady-state errors for Case 2. Although selecting smaller control gains can diminish the oscillations of tracking errors in Case 1, the insignificant control gains may increase the steady-state tracking errors in Case 2. Subsequently, the FOPID controller was reapplied to the VCM control system and the corresponding experimental results are shown in Figure 7. As seen from Figure 7c, the FOPID controller was able to construct more effect control signals for restraining the oscillations of tracking errors effectively. Besides, the tracking errors were substantially improved as shown in Figure 7d in Case 2. However, the tracking errors remained unfavorable owing to the fixed control gains ineffectively addressing the external disturbance and uncertainties.

The proposed SFOPID controller was applied to the VCM control system finally in which the parameters *k_p_*, *k_i_*, *k_d_*, α and β are self-tuning between the 1st and 2nd seconds by using the ADE algorithm. The constant parameters for the ADE algorithm were chosen as *NP* = 5, Φ = 5, *N_q_* = 5, *N_g_* = 40, *CR* = 0.4, *q_d_* = 0.2, λ*_b_* = 1.2, and λ*_s_* = 0.8. Regarding the practical requirements, the lower bounds for the control parameters were designed as *k_p_*_min_ = 5, *k_i_*_min_ = 25, *k_d_*_min_ = 1.5, α_min_ = 0.3, and β_min_ = 0.3 while the upper bounds were designed as *k_p_*_max_ = 20, *k_i_*_max_ = 50, *k_d_*_max_ = 2.5, α_max_ = 0.7, and β_max_ = 0.7, respectively. The initial five target vectors were randomly generated within the specific ranges. 

**Figure 8:**
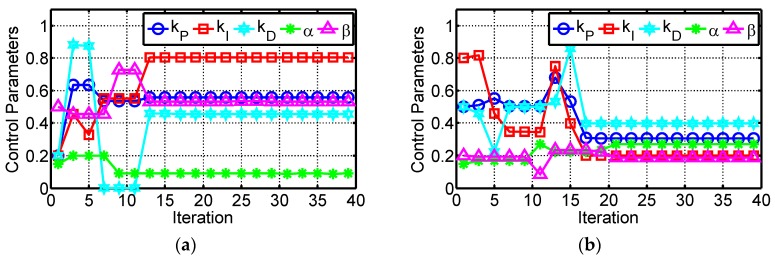
Evolutions of the self-tuning control parameters: (**a**) normalized values in Case 1; and (**b**) normalized values in Case 2.

**Figure 9:**
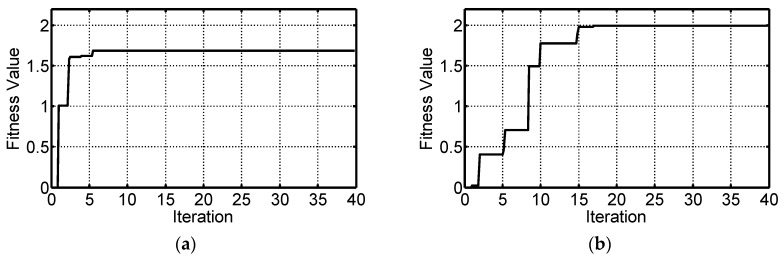
Evolutions of the fitness values optimized by ADE algorithm: (**a**) fitness values in Case 1; and (**b**) fitness values in Case 2.

To show the evolutions of the self-tuning control parameters, the movements of the first individual vector in the population are shown in Figure 8. It is noted that all of the values were normalized to [0,1] for clear illustration. In the evolutions, all of the target vectors had random values initially and searched for the optimal solutions individually, as shown in Figure 8. The effectiveness of the ADE algorithm was demonstrated by the gradually increased fitness value. The evolution of the fitness value for the first individual vector is shown in Figure 9. After the 6th iteration in Case 1 and 17th iteration in Case 2, the fitness values were stable which indicated that all of the optimal adaptive control parameters were found. The eventual optimized control parameters of *k_p_*, *k_i_*, *k_d_*, *α* and *β* are 8.07, 29.33, 1.90, 0.37, and 0.41 in Case 1 and 10.58, 37.39, 1.95, 0.51 and 0.34 in Case 2, respectively.

**Figure 10:**
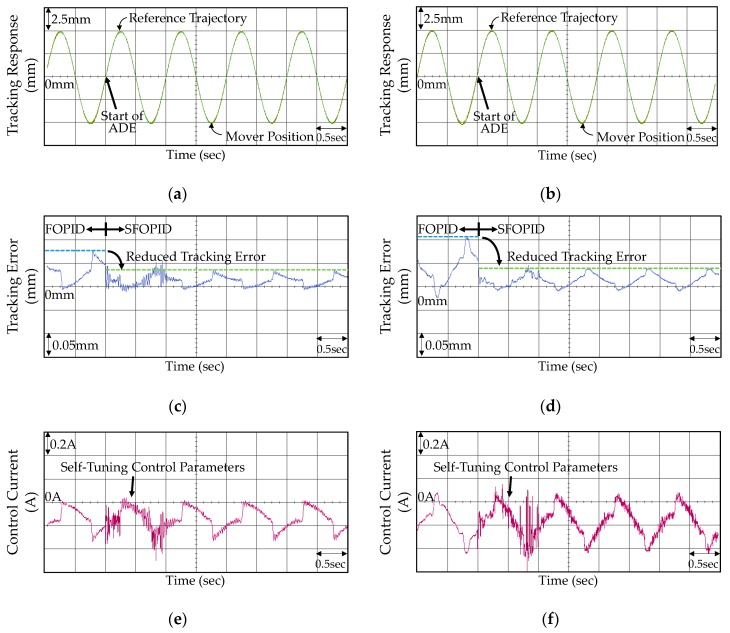
Experimental results of the VCM control system using the SFOPID controller: (**a**) tracking response in Case 1; (**b**) tracking response in Case 2; (**c**) tracking error in Case 1; (**d**) tracking error in Case 2; (**e**) control current in Case 1; and (**f**) control current in Case 2.

The experimental results of VCM control system using the proposed SFOPID controller are shown in Figure 10. As seen from Figure 10c,d, the VCM is controlled by the FOPID controller in the first one second, and controlled by the SFOPID controller thereafter. Though the control parameters of the FOPID controller were selected by several trials to achieve the favorable transient control performance levels regarding the steady-state stability, the maximum and average tracking errors in both nominal and payload conditions are both reduced effectively from the 2nd second. These facts reveal that the optimized control parameters are able to improve the tracking performances in practical control applications. From the experimental results shown in Figure 6, Figure 7, and Figure 10, the best control performance of the SFOPID controller for both nominal and payload conditions can be clearly observed.

To evaluate the control performances of the various VCM control systems, the performance measures and improvement rates of the PID, FOPID, and SFOPID controllers in Cases 1 and 2 were compared in Table 1 and Table 2. As seen from Table 1 and Table 2, the performance measures *P_M_*, *P_A_*, and *P_S_* of the PID controller were markedly reduced by the FOPID controller with respect to the two well-defined fractional orders. Moreover, the proposed SFOPID controller further improves the tracking performances of the FOPID controller because all the control parameters were globally and dynamically optimized by means of the ADE algorithm.

**Figure 11:**
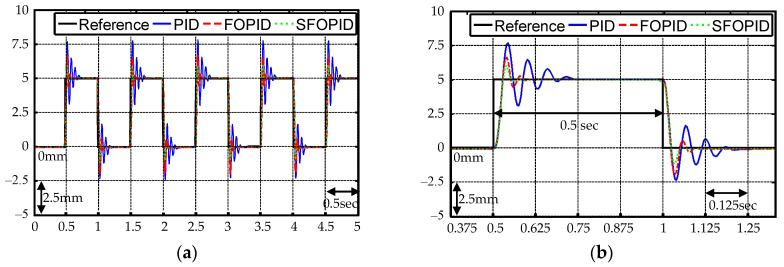
Step responses of the VCM control system using the PID, FOPID, and SFOPID controllers: (**a**) complete step responses; (**b**) first two step responses.

To further demonstrate the improved control performance of the proposed SFOPID controller, continuous step references’ tracking is tested experimentally. The experimental results including complete step responses and the first two step responses are shown in Figure 11. As seen from Figure 11, poor tracking responses such as large maximum overshoot and long settling time were obtained by the PID controller. Though the selection of smaller control parameters can reduce maximum overshoot and settling time, the robustness will be exacerbated accordingly in the steady-state. Moreover, the tracking performances were improved apparently by the FOPID controller, which reveals that the addition of two well-defined fractional differential and integral orders is able to construct more effective control signals for suppressing the tracking error. Finally, the designed SFOPID controller was reapplied to the VCM system. The relevant experimental results indicate that the tracking errors were markedly decreased through the online optimization of control parameters. Moreover, the corresponding maximum overshoot *M_o_*, average tracking error *P_A_*, and settling time *T_s_* summarized in Table 3 show that the proposed SFOPID controller can significantly improve the control performances of the PID and FOPID controllers with regard to the nonlinear VCM control system.

## 6. Conclusions

This study demonstrated the design and implementation of the SFOPID controller with ADE optimization for the high precision position control of a linear VCM control system. First, the operational principle and dynamics of the VCM system were described. Then, the theoretical base of the fractional calculus was given. Subsequently, the detailed control system design strategy of the proposed SFOPID controller was introduced. In the SFOPID controller, an ADE algorithm with an adaptive selection mechanism was adopted to optimize the five control parameters for the minimization of position error online. Finally, the experiments were conducted using a digital embedded control platform in which the fractional order calculus was performed by the Tustin discretization approximation method. Experimental results with performance measures indicate that the proposed SFOPID improves the tracking performances of the PID and FOPID with regard to the VCM under different operating conditions significantly. Thus, the major contributions of this study can be summarized as: (i) the successful development of the new SFOPID controller which optimizes the conventional FOPID controller online; and (ii) the successful implementation and comparison of the PID, FOPID, and SFOPID controllers for the VCM control system. Moreover, to design the control parameters for the PID and FOPID controllers and initial target vector for the SFOPID controller more effectively, the system parameters identification for the VCM will be conducted in the future work.

## Figures and Tables

**Table 1:** Performance measures and improvement rates for the sinusoidal reference tracking of various VCM control systems in Case 1.

Controllers	Performance Measures (μm)			Improvement Rates (%)		
	*P_M_*	*P_A_*	*P_S_*	*P_M_*	*P_A_*	*P_S_*
PID	105	42	33	Baseline	Baseline	Baseline
FOPID	78	31	20	25.71	26.19	39.39
SFOPID	35	13	9	66.67	69.05	72.73

**Table 2:** Performance measures and improvement rates for the sinusoidal reference tracking of various VCM control systems in Case 2.

Controllers	Performance Measures (μm)			Improvement Rates (%)		
	*P_M_*	*P_A_*	*P_S_*	*P_M_*	*P_A_*	*P_S_*
PID	142	55	37	Baseline	Baseline	Baseline
FOPID	106	42	29	25.35	23.64	21.62
SFOPID	39	16	11	72.54	70.91	70.27

**Table 3:** Performance measures and improvement rates for the continuous step responses of various VCM control systems.

Controllers	Performance Measures			Improvement Rates (%)		
	*M_o_* (mm)	*P_A_* (mm)	*T_s_* (sec)	*M_o_*	*P_A_*	*T_s_*
PID	2.69	0.42	0.227	Baseline	Baseline	Baseline
FOPID	1.65	0.22	0.085	38.66	47.62	62.56
SFOPID	1.03	0.19	0.046	61.71	54.76	79.74
